# Semi-Supervised Learning of Cartesian Factors: A Top-Down Model of the Entorhinal Hippocampal Complex

**DOI:** 10.3389/fpsyg.2017.00215

**Published:** 2017-02-21

**Authors:** András Lőrincz, András Sárkány

**Affiliations:** Neural Information Processing Group, Faculty of Informatics, Eötvös Loránd University Budapest, Hungary

**Keywords:** Cartesian factors, entorhinal hippocampal complex, integrate-and-fire neurons, head direction cells, place cells, grid cells, border cells

## Abstract

The existence of place cells (PCs), grid cells (GCs), border cells (BCs), and head direction cells (HCs) as well as the dependencies between them have been enigmatic. We make an effort to explain their nature by introducing the concept of Cartesian Factors. These factors have specific properties: (i) they assume and complement each other, like direction and position and (ii) they have localized discrete representations *with* predictive attractors enabling implicit metric-like computations. In our model, HCs make the distributed and local representation of direction. Predictive attractor dynamics on that network forms the Cartesian Factor “*direction.”* We embed these HCs *and* idiothetic visual information into a semi-supervised sparse autoencoding comparator structure that compresses its inputs and learns PCs, the distributed local and direction independent (allothetic) representation of the Cartesian Factor of global space. We use a supervised, information compressing predictive algorithm and form direction sensitive (oriented) GCs from the learned PCs by means of an attractor-like algorithm. Since the algorithm can continue the grid structure beyond the region of the PCs, i.e., beyond its learning domain, thus the GCs and the PCs *together* form our metric-like Cartesian Factors of space. We also stipulate that the same algorithm can produce BCs. Our algorithm applies (a) a bag representation that models the “what system” and (b) magnitude ordered place cell activities that model either the integrate-and-fire mechanism, or theta phase precession, or both. We relate the components of the algorithm to the entorhinal-hippocampal complex and to its working. The algorithm requires both spatial and lifetime sparsification that may gain support from the two-stage memory formation of this complex.

## 1. Introduction

The fact that we are able to describe autobiographic events, can discover rules, in spite of the many dimensional inputs, such as the retina (millions of photoreceptors), the ear (cca. 15,000 inner plus outer hair cells), the large number of chemoreceptors as well as proprioceptive, mechanoreceptive, thermoceptive and nociceptive sensory receptors is puzzling, since the number of sensors enters the exponent of the size of the state space. This number is gigantic even if the basis of exponent is only 2, but it is typically much larger. How is it possible to remember for anything in such a huge space?

An illuminating and classical observation has been made by Kohonen (1982): the brain develops low dimensional representations, sometimes in the form of topographic maps manifested by retinotopy in the visual system, tonotopy in the auditory system, somatotopy in the somatosensory system, and so on. Kohonen considered these maps as some kind of *implicit* metric of the sensed space, being visual, auditory, or body related. The dimensionality of these maps is low, unlike the number of the sensors that give rise to those maps. Similarly low-dimensional representations of *the external space* appear in the entorhinal-hippocampal complex (EHC), although the topography is sometimes missing. The derivation of the abstract and low-dimensional representation of space from the actual and high dimensional sensory information is critical for goal oriented behavior as noted in the context of reinforcement learning, see, e.g., Kearns and Koller (1999), Boutilier et al. (2000), and Szita and Lőrincz (2009). In this context, one is directed to the EHC. The importance of this complex was discovered many years ago by Scoville and Milner (1957). Now, it is widely accepted that the EHC is responsible for episodic memory, see, e.g., Squire and Zola (1998) and Moscovitch et al. (2016) for an earlier review and for a recent one, respectively. In their paper, Buzsáki and Moser (2013) propose that (i) planning has evolved from navigation in the physical world, (ii) that navigation in real and mental space are fundamentally the same, and (iii) they underline the hypothesis that the EHC supports navigation and memory formation.

We believe that one of the functional tasks of this complex is the learning of low-dimensional Cartesian Factors that we define as follows. We say that (i) a low-dimensional representation discretizes a low dimensional variable, if discretization means that individual neurons [e.g., *place cells* (PCs) discovered more than 40 years ago (O'Keefe and Dostrovsky, 1971; O'Keefe and Nadel, 1978) represent local regions of their space (the so called place fields for PCs)] and thus the representation of the variable is distributed, (ii) the variable could be used as a coordinate in controlling and cognitive tasks, and (iii) an attractor network can predict by means of the local representation and, in turn, it can work as an implicit metric. As a further specification, we distinguish two factor types. Components of the first kind may exist even if other ones are not present, whereas components of Cartesian Factors do assume each other; no Cartesian Factor may exist without the others although many of them can be latent. We detail this below:

- Type I factors make no (or minor) assumptions about each other. Non-negative matrix factorization (NMF), for example, originates from chemistry: it is used in mass spectrometry and radiology among other fields, where absorbing or radiating components can sum up. In a given environment and for a given detector system, the observation of different isotopes depends on the environment and the detector, but they do not influence each other's spectrum except that—to a good approximation—they sum up. Another example is slow vs. faster or fast features (Franzius et al., 2007; Schönfeld and Wiskott, 2015). Such Type I factors are called features in most cases; they can be independent, one of them may not have to imply the presence of others. In other words, if one of the NMF or slow feature components is present, others can be missing.
- Type II factors assume each other. For example, texture, shape, weight, material components belong to the same object and any object possess all of these features. Some of them can be relevant when considering the value of a tool in a task. Another example is the information about the position of an object in space that can be given by the spatial coordinates and its pose. The speed of the object is another component, being necessary for the characterization of its state in certain tasks.

Latent Type II factors can serve cognition by decreasing the description and thus the state space. Keeping the example of the space, path planning requires the discretization of space and information about the neighboring relations of the PCs, i.e., the neighbor graph. Then an algorithm can find the shortest path on the graph. This path planning procedure doesn't require directional information; it works in a reduced dimensional space. We are concerned with such complementing and dimension reducing factors that may alleviate cognition in different ways in different tasks.

We assume that there is at least one Type II factor that can be sensed directly and this factor is represented in a topographic manner: it has some kind of (implicit) metric. This factor plays the role of a *semi-supervisor* in the learning of the complementing Type II factor(s). We also assume that the complementing factor is also low dimensional. Allothetic representation of the space is one example of such factors and it is the complementing factor of the allothetic representation of direction. *Head direction cells* (HCs) (see e.g., the work of Taube, 2007 and the references therein) make the discretized allothetic head direction representation. An attractor network can predict the activity pattern of the representation during rotation making it an (implicit) metric-like representation of direction. In turn, the set of HCs make a *Cartesian Factor*. We will consider how a metric-like representation may emerge from neurally plausible dynamics and the PC representation via predictive methods.

We note that according to Winter et al. (2015), in rodents, HCs are needed for the development of PCs, which are localized (i.e., discretized and distributed) allothetic representation of space; Type II factor according to our concepts.

There are neurons that respond along trigonal grids. These are the so called *grid cells* (GCs) (Fyhn et al., 2004; Moser et al., 2014). Results of Bonnevie et al. (2013) indicate that the presence of GCs is conditioned on both the presence of PCs *and* on the availability of HCs. For a recent review of the grid cells and the place cells see, e.g., the collection edited by Derdikman and Knierim (2014) as well as the references cited therein.

Our contributions are as follows.

1. We present a unified model of the EHC. We put forth the idea that this complex tries to solve the problem of nonlinear dimensionality reduction via Type II factors. These reduced dimensions function are like Cartesian coordinates if attractor networks enable them to form an implicit metric. Such Cartesian Factors can be reasoned with like symbolic variables. Consequently, we see the continuation of the grid as *learnable manipulation* at the symbolic level called *mind travel* by Sanders et al. (2015). The grounding of the symbolic manipulation beyond the known domain seems as a necessity for acting according to Harnad (1990). A simple example is *homing behavior*; the transformation of goals in allothetic PCs to idiothetic action series.
2. The model is a learning model, which is capable of explaining (a) peculiar findings on the inter-dependencies of PCs and GCs, including (b) the corruptions that occur upon lesioning of different components and (c) the order of learning as described in the recent review paper of Rowland et al. (2016).
3. Direction sensitive GCs are developed from PCs and HCs by means of a predictive and compressing supervised algorithm working on *magnitude ordered neural activities*. We argue that either (a) integrate-and-fire characteristics or (b) theta phase precession can give rise to magnitude ordering in the time domain. We apply two simple linear algorithm on the ordered representation; we use *pseudoinverse computation* and *partial least squares* (PLS) regression. We show that PLS regression produces orientation sensitive, close to hexagonal grids in an incommensurate squared environment. We demonstrate that magnitude ordered predictive grid representation can be continued beyond the experienced environment.
4. We show that the predictive mechanism that gives rise to direction sensitive GCs can support the learning of Border Cells (BCs).
5. Our autoencoder model exploits sparsification and has the following constraints: we find that *lifetime sparsification*, i.e., sparsification over a larger number of inputs is necessary for efficient learning. Lifetime sparsification is not possible in real time, when individual input based sparsification, called *spatial sparsification* is needed. We propose that the two types of sparsification may be (one of) the underlying reason(s) of the two-stage memory formation in the EHC loop (Buzsáki, 1989).

Cartesian Factors have been introduced in two previous conference papers (Lőrincz et al., 2016; Lőrincz, 2016). The definition presented here is more precise and more elaborate: *Cartesian Factors complement each other and assume metric-like representations*. PCs have been developed in those publications and we review the results here. The extension of the model with orientation sensitive grid cells appears here for the first time alike to the proposal that magnitude ordered representation can serve the learning. Both integrate-and-fire behavior and theta phase precession are neurally plausible mechanism for magnitude ordering. In the first case, the spike representing the highest magnitude input comes first. In the second case, highest firing rates occur in the middle of the theta cycles. The combined model of direction sensitive GCs, PCs, and BCs is presented here for the first time.

In the following sections, we review background information and list some of the models of place cell and grid formation (Section 2). We describe the algorithmic components of our model in Section 3. More details of the algorithms are provided in the Appendix. The results section (Section 4) presents PC and directional sensitive GC representations. Results are discussed from the point of view of neuroscience in Section 5. We also consider symbolic representation, symbol manipulation and the symbol grounding problem in this section. We argue that all components—i.e., Cartesian Factors, place cell forming algorithms, oriented grid learning computational methods, and border cell formation—may fit the features of the EHC. Conclusions are drawn in Section 6.

## 2. Background

### 2.1. Review of related findings in the EHC

The set of PCs, also called the *cognitive map*, the orientation independent representation of space, was discovered more than 40 years ago (O'Keefe and Dostrovsky, 1971; O'Keefe and Nadel, 1978). Since then we have learned many features of these cells, which are present in the CA3 and CA1 subfields of the hippocampus. Theta frequency oscillations (5–10 Hz) in the rodent hippocampal system create theta sequences: (i) place cells fire in temporal order, (ii) the sequences cover past, present and future, and (iii) time compression can be as much as a factor of 10 (Skaggs and McNaughton, 1996). Such temporal series centered on the present are the so called (theta) phase precession of PCs. The CA3 subfield has a recurrent collateral structure that, during sharp wave ripple (SPW-R, 140–200 Hz) complexes, replays temporal series experienced during exploratory behavior, when theta oscillations occur. Time series compression in SPW-R is around twenty fold and forty fold, before and after learning, respectively as shown by Lee and Wilson (2002). Memory trace formation seems to require to stages, the theta-concurrent exploratory activity and the population burst during SPW-R following the explorations (Buzsáki, 1989; Chrobak and Buzsáki, 1994) and according to the widely accepted view, the EHC formed memories include episodic ones (Moscovitch et al., 2016). The hippocampal formation is needed for dead reckoning (path integration) (Whishaw et al., 2001).

Grid cells have been found in the medial entorhinal cortex (MEC). It turns out that MEC lesion can abolish phase precession (Schlesiger et al., 2015; Wang et al., 2015), but the lesion only corrupts hippocampal place cells, it can't fully eliminate them (Hales et al., 2014). On the other hand, grid cells require hippocampal input (Bonnevie et al., 2013). The excellent review of Sanders et al. (2015) about place cells, grid cells, and phase precession includes a novel model about the two halves, i.e., about the past and the future. They claim that different mechanisms operate during the two halves.

Another important feature is that both the grid representation in the entorhinal cortex and the place cell representation of the hippocampus depend strongly on the vestibular information. There are indications put forth by Winter and Taube (2014) that head direction cells may not be critical for place cell formation since those can be controlled by environmental cues, like visual landmarks. However, it was shown by Winter et al. (2015) that the disruption of head direction cells can impair grid cell signals and are crucial for the formation of the allothetic representation including both place cells and grid cells. They also reported that theta waves are spared upon the same manipulation.

We shall argue that several findings follow from the constraints of developing the Cartesian Factor abstraction and the related metric-like representations.

### 2.2. Related models

The number of place cell models is considerable, we list only a few of them. The interested reader is directed to the recent publication of Schultheiss et al. (2015) that reviews both mechanistic bottom-up models and top-down models.

Neural representation of trajectories traveled and the connectivity structure developed during such paths have been suggested as the method for place cell formation by Redish and Touretzky (1998). Incoming information includes external cues and internally generated signals. They are fused to develop place cells in the paper of Arleo and Gerstner (2000). Place cells were derived by Solstad et al. (2006) from linear combinations of entorhinal grid cells (Fyhn et al., 2004) and vice versa, neuronal level model can produce grid cell firing from place cell activities as shown by Burgess and O'Keefe (2011). Time plays the key role in the slow feature analysis model of place cells put forth by Franzius et al. (2007) and Schönfeld and Wiskott (2015). Time plays the opposite role in the independent component analysis based autoencoding place cell models (Lőrincz and Buzsáki, 2000; Lőrincz and Szirtes, 2009). In these works, time appears in a so called novelty detection (time differentiation) step.

We think that all of these models, i.e., navigation based models, models based on interaction between representations, models that search for components that change slowly in time, and models that consider novelty detection may have their merits in the development of low-dimensional representation of Cartesian Factors, since the development os such representations—as it has been mentioned earlier—are crucial for reinforcement learning of goal oriented behavior. For example, navigation in partially observed environments, like the Morris maze or when in dark, can be supported by temporal integration. As another example, novelty detection may support the separation of a rotating platform from remote, non-rotating cues studied by the Stuchlik group (Stuchlik and Bures, 2002; Stuchlik et al., 2013). Further, the relevance of learning of low-dimensional task oriented representations can't be underestimated since state space and thus learning time decreases tremendously if the dimension is decreased.

It seems straightforward to us that information both from the environment and from self-motion should be combined for an efficient and precise neural representation of self motion in the external space (Evans et al., 2016) and that different signals and latent variables can be advantageous under different conditions and may support each other. The case is similar to object recognition, when the different mechanisms, such as stereoscopic information, structure from motion, shape from shading, texture gradient, and occlusion contours among others work together in order to disambiguate the “blooming, buzzing confusion” of the visual information in different conditions, see, e.g., the work of Todd (2004) and the references in that paper.

Due to the critical nature of the vestibular input, our goal is to derive place cells under the assumption that only this component of the Cartesian representation, namely the egocentric direction relative to an allothetic coordinate system is available and we ask if the allothetic representation of space can be derived by using only (i) directional information and (ii) the egocentric, i.e., idiothetic visual information.

## 3. Review of the algorithms

### 3.1. The logic of the algorithmic components

The logic is as follows:

- We start with an autoencoding network and meet the comparator hypothesis of Vinogradova (2001).
- Firing in the hippocampus is very sparse, see, e.g., the work of Quiroga et al. (2008), and we apply sparse models.
- We find limitations and include lifetime sparsity beyond the spatial one. It is supported by the two-stage formation of memory traces.
- We derive the dynamics of the grid structure by predicting in the simplest form: input–output pairs are formed by past and future experiences, respectively. The predicted values can be fed back, the input can be shifted by them and thus, prediction can be continued into the future. We compare linear models; the pseudoinverse computation and partial least square regression.
- Prediction concerns the actual firing pattern instead of the individual neurons that fire and components are ordered by their magnitudes: the largest magnitude signal makes the first component of the input and so on in decreasing order. This feature may appear naturally in integrate-and-fire mechanisms.
- We assume view invariant observations of the objects. We use indices: a visible object activates an index. This is like the recognition of the presence of the object (“what”) without the knowledge about its position (“where”). This “what” representation resembles to the so called “bag model” (Harris, 1954; Csurka et al., 2004).

Below, we elaborate on these algorithms and then we present our results.

### 3.2. Autoencoder

An autoencoder is the self-supervised version of the Multilayer Perceptron (MLP) and may have *deep* versions (Hinton and Salakhutdinov, 2006; Vincent et al., 2010). For the sake of general formulation, the deep version is described below although our numerical studies in this respect are limited.

Consider a series of non-linear mappings (layers) of the form:


(1) $$\begin{array}{l} H=f_{N}\left(\cdots f_{2}\left(f_{1}\left(XW_{1}\right)W_{2}\right)\cdots W_{N}\right), \end{array}$$

where ***X*** ∈ ℝ^*I* × *J*^ is the matrix of *I* inputs of size *J*, ***W***_*n*_ ∈ ℝ^*Q*_*n*−1_,*Q*_*n*_^ are parameters with *Q*_0_ = *J*, and *f*_*n*_ are non-linear almost everywhere differentiable element-wise functions (*n* = 1, …, *N*; *N* ∈ ℕ). Then ***H*** ∈ ℝ^*I* × *Q*^ is called the feature map (*Q*_*N*_ = *Q*). Typically, one takes two such mappings with reversed sizes—an encoder and a decoder—and composes them. Thereupon one can define a so-called reconstruction error between the encoder input ***X*** and the decoder output $\hat{X}\in {\mathbb{R}}^{I\times J}$, normally the ℓ_2_ or Frobenius norm of the difference, i.e.,


$$\begin{array}{l} \frac{1}{2}||X-\hat{X}|{|}_{F}^{2}=\frac{1}{2}\underset{i=1,\ldots ,I}{\sum }\underset{j=1,\ldots ,J}{\sum }{\left(X_{i,j}-{\hat{X}}_{i,j}\right)}^{2} \end{array}$$

and try to find a local minimum of it in terms of parameters ***W***_*n*_ after random initialization, by taking advantage of a step-size adaptive mini-batch subgradient descent method (Duchi et al., 2011; Zeiler, 2012; Kingma and Ba, 2014). The non-linearity can be chosen to be the rectified linear function *f*_*n*_(*x*) = *x* · *I* (*x* > 0) for *x* ∈ ℝ (Nair and Hinton, 2010; Dahl et al., 2013) to avoid the vanishing gradient problem (Hochreiter, 1991; Hochreiter et al., 2001), where *I* designates the indicator function.

### 3.3. Spatial sparsity and lifetime sparsity

Deep Autoencoders are often used as a pretraining scheme, see, e.g., the work of Erhan et al. (2010), or as a part of supervised algorithms as in the paper of Rasmus et al. (2015), but they lack the ability to learn a meaningful or simple data representation without prior knowledge (Sun et al., 2017). To obtain such a description, one might add regularizers or constraints to the objective function as did Grant and Boyd (2014) and Becker et al. (2011), or employ a greedy procedure like Tropp and Gilbert (2007) and Dai and Milenkovic (2009). It is well known that minimizing the sum of ℓ_2_ norms of parameters ***W***_*n*_ can reduce model complexity by yielding a dense feature map, and similarly, the ℓ_1_ variant may result in a sparse version (Tibshirani, 1996; Ng, 2004).

An alternative possibility is to introduce constraints in the non-linear function *f*_*n*_. For example, one may utilize a *k*-sparse representation by keeping solely the top *k* activation values in feature map ***H***, and letting the rest of the components zero as suggested by Makhzani and Frey (2013). This case, when features, i.e., the components of the representation, compete with each other is referred to as *spatial sparsity*.

Sparsification occurs on a different ground if indices of the representation on *many* inputs go up against each other. This case is called *lifetime sparsity*, see, e.g., the work of Makhzani and Frey (2015) and the references therein. Lifetime sparsification ensures that all indices may play a role, whereas spatial sparsification may render a large portion of the components of the representation quiet for all inputs. On the other hand, lifetime sparsification may not be used on any single input, the case needed for real time responses.

### 3.4. Predictive partial least squares regression

PLS regression started with the works of Kowalski et al. (1982) and Geladi and Kowalski (1986) back in the eighties. The PLS model assumes explanatory samples collected in matrix ***R*** made of *t* samples of *l* dimensions and a response matrix ***Q*** of *m* dimensions collected on the *t* observations. PLS combines features of principal component regression (PCR) and multiple linear regression (MLR): PCR finds maximum variance in ***R***, MLR is to maximize correlation between ***R*** and ***Q***. PLS regression tries to do both by maximizing covariance between them: first, it extracts a set of latent factors that explain the covariance between the explanatory and response variables and then the regression step predicts the values of the response variables.

In our case, explanatory variables and responses are connected by time: ***R*** = [***r***(1), …, ***r***(*t*)] and ***Q*** = ***R***(+) = [*r*(2), …, *r*(*t*+1)] make the explanatory and the response variables, respectively. PLS regression takes the form


(2) $$\begin{array}{r} R=TP^{T}+E \end{array}$$

(3) $$\begin{array}{r} R\left(+\right)=UQ^{T}+F \end{array}$$

where ***T*** and ***U*** are matrices of dimensions *t* × *n*, ***P*** and ***Q*** are the so called orthogonal loading matrices of dimensions *t* × *n* (***P***^*T*^***P*** = ***Q***^*T*^***Q*** = *I*), and matrices ***E*** and ***F*** are the error terms drawn from independent and identically distributed random normal variables. It is also assumed that covariance between matrices ***T*** and ***U*** are maximal. In the computations, we used the Python package *sklearn* (Pedregosa et al., 2011).

### 3.5. Prediction via pseudoinverse computation

PLS regression is one option for predictions. Deep networks can be considerably more efficient. The simplest method, on the other hand, is possibly pseudoinverse computation that can be embedded into a Hebbian network structure as suggested by Lőrincz and Szirtes (2009) and in some of the references cited. Using the notations of the previous section, the pseudoinverse solution can be formulated as follows:


(4) $$\begin{array}{l} r\left(\tau +1\right)=M{\left(r{\left(\tau \right)}^{T},\ldots ,r{\left(\tau -t\right)}^{T}\right)}^{T}+e\left(t\right) \end{array}$$

where *e*(*t*) is the error term at time *t*. Equation (4) gives rise to the solution $\hat{M}\approx R\left(+\right){ℜ}^{+}$ where ℜ^+^ denotes the Moore–Penrose right pseudoinverse of the matrix constructed from the matrix with the *i*^*th*^ column formed by (***r***(*i*)^*T*^, …; ***r***(*i*−*t*)^*T*^)*T* and *i* > *n* is assumed.

#### 3.5.1. Continued prediction

For the pseudoinverse method, matrix $\hat{M}$ and the estimated predicted activities can be used for shifting the prediction further in time


(5) $$\begin{array}{r} \hat{r}\left(\tau +1\right)\approx \hat{M}{\left(r{\left(\tau \right)}^{T},\ldots ,r{\left(\tau -t\right)}^{T}\right)}^{T} \end{array}$$

(6) $$\begin{array}{l} \;\hat{r}\left(\tau +2\right)\approx \hat{M}{\left(\hat{r}{\left(\tau +1\right)}^{T},\ldots ,r{\left(\tau -t+1\right)}^{T}\right)}^{T}\\ \text{and so on} \end{array}$$

and the case is similar for the PLS regression.

### 3.6. Magnitude ordered activities

PC activities themselves are bounded to the PCs themselves. This representation can't fulfill our purposes since PCs are locked to already observed bag representations and thus they are not able to support prediction outside of the explored field. As we shall see, sparse autoencoder on the bag representation produces densely packed PCs that have high activities at the centers and lower activities off-center. In turn, between two place cell bumps there should be a hump and a metric-like representation can take advantage of this periodicity. If we order activities according to their magnitudes then largest activity will reach its (local) maximum at the center of a place cell, it will be smaller at other (neighboring) positions and will become large at another center. We will develop latent predictive factors of the magnitude ordered place cell activities. Indications that magnitude based ordering may be present in the neural substrate is elaborated in the discussion (Section 5.2).

### 3.7. The bag model

We assume a high level representation of the visual information that correspond to the so called bag model of machine learning. The Bag of Words representation, for example, represents a document by the words that occur in the document, without any syntactic information (Harris, 1954). Similarly, the Bag of Keypoints representation of an image (see e.g., Csurka et al., 2004 and the references therein) contains the visual descriptors of the image without any information about the position of those descriptors. Such representations are similar to the *what system* in visual processing as described first by Mishkin and Ungerleider (1982), elaborated later by Goodale and Milner (1992) and that may also be present in the representations of other modalities, see, e.g., the work of Schubotz et al. (2003).

Our inputs are represented by the indices of the objects present in the visual field. If the object is present, then the value at corresponding input component is set to 1. Otherwise, it is set to zero. This representation is independent from the position of the object within the visual field, being an invariant representation of the object, since the value of the representation does not change as a function of idiothetic direction and allothetic position as long as the object is within the visual field.

### 3.8. Algorithmic formulation of Cartesian Factor learning

**Figure 1:**
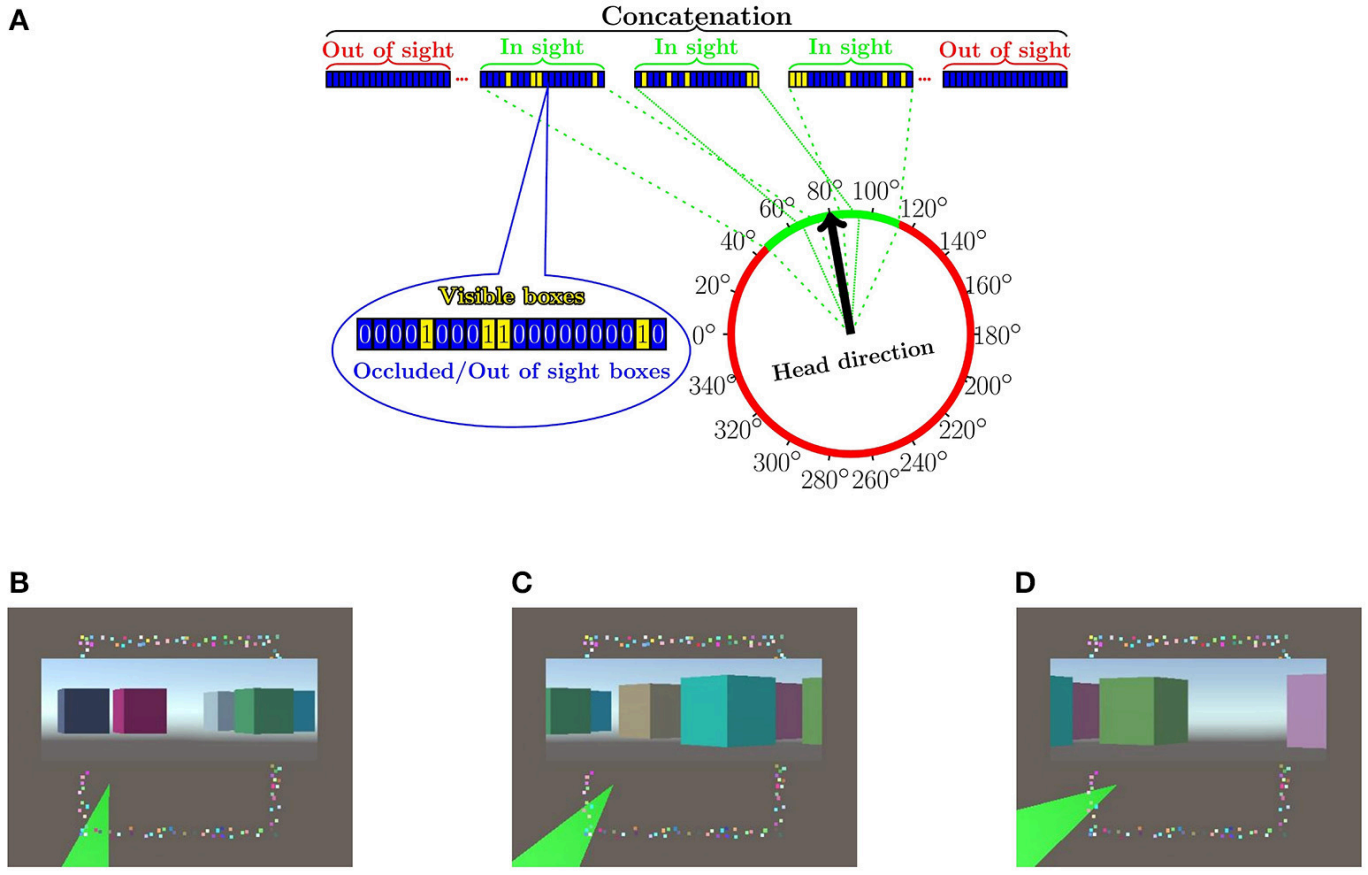
**Arrangements of the numerical experiments. (A)** Input is concatenated from sub-vectors, which belong to different allothetic directions. A given index corresponds to the same box, the “remote visible cue,” in all sub-vectors. The value of the a component of a sub-vector is 1 (0) if the box is visible (non-visible) in the corresponding direction (cf. bag representation, for more details, see text). More than one direction can be visible. The figure shows the case of three visible directions depicted by green color. Some boxes may be present in more than one visible direction, since they are large. **(B–D)** The “arena” from above with the different boxes around it plus some insets. Shaded green areas in **(B–D)**, show the visible portions within the field of view at a given position with a given head direction. Insets show the visual information for each portion to be transformed to 1 s and 0 s in the respective components of the sub-vectors. Components of out-of-view sub-vectors are set to zero. (Lőrincz et al., 2016 with permission).

We assume that a latent random variable *Z* (e.g., the discretized allothetic representation of the state, that is, the place cells) and an observed random variable *Y* (e.g., the head direction, that is, a compass) are continuous and together they can fully explain away—by means of saved memories—another observed binary random variable *X* (e.g., the egocentric view with pixel values either one or zero taken in the direction of the head, or the invariant bag representation with ones and zeros). The ranges of *Z* and *Y* are supposed to be discretized finite *r*- and one-dimensional intervals, respectively. For more details, see Figure 1 and the Appendix.

### 3.9. Simulation environment and numerical details

#### 3.9.1. The arena

For our study, we generated a squared “arena” surrounded by *d* = 150 boxes (Figure 1). The “arena” had no obstacles. Boxes were placed pseudo-randomly: they did not overlap. The “arena” was discretized by an *M* × *M* = 36 × 36 grid. From each grid point and for every 20°, a 28° field of view was created (i.e., $L=\frac{360^{o}}{20^{o}}=18$, overlap: 4° between regions), and the visibility—a binary value (0 for occlusion or out of the angle of view)—for each box was recorded, according to Equation (7); we constructed a total of *I* = 37·37·18 = 24, 642 binary (***x***^(*m, l*)^) vectors.

#### 3.9.2. Masks and information on closeness

**Figure 2:**
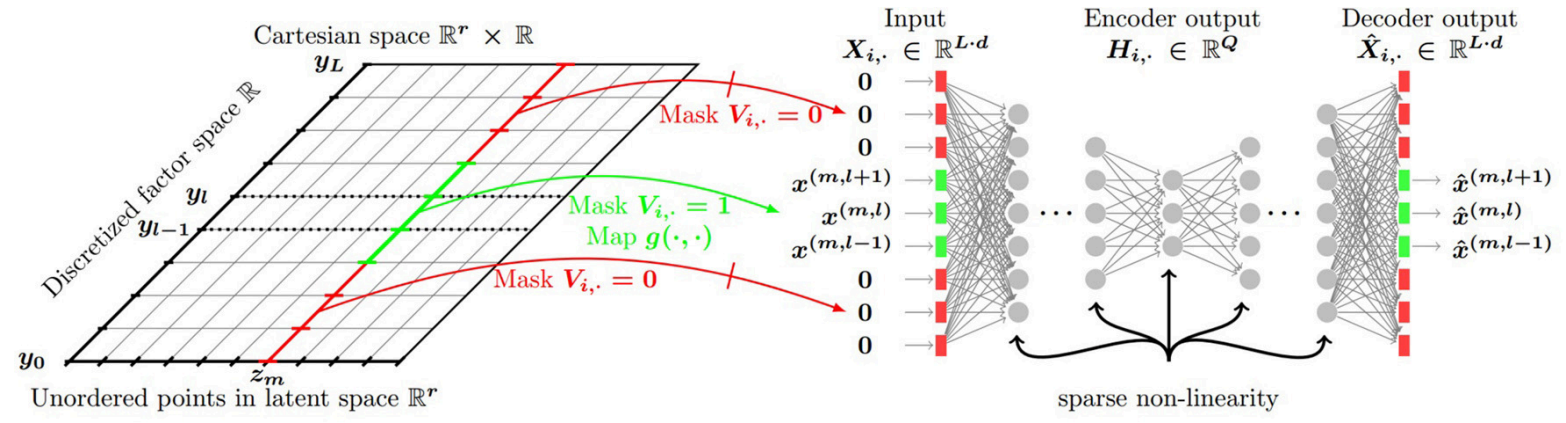
**General architecture**. In the numerical experiments the notations correspond to the following quantities: *Z* latent positions, *Y* discretized “compass” values. Non-visible part of the input to the network is denoted by red, visible part is denoted by green. Visible part consists of 28° viewing angle in the actual direction and 28° viewing angle in neighboring directions separated by 20°. The number of neighbors was set to 2, 4, 16, 17 with 17 directions and the actual direction covering the whole 360°. For each viewing angle inputs represent boxes visible within the corresponding range. Values of the vector components representing the boxes are set to 1 if the range corresponds to the actual direction or if belongs to the set of neighbors. The full size of the input equals to the “No. of boxes × No. of viewing angle ranges.” (Lőrincz et al., 2016 with permission).

These vectors were processed further. Beyond the actual viewing direction and viewing angle of 28°, we also input visual information in neighboring directions: we varied the non-zeroed (non-masked) part of the input from a single direction (28°) to all 18 directions (360°). Formally, for various experiments, we defined masks ***V***_*i*, ·_ summing to *v* = 1, 3, …, 17, 18, for which we carried out the concatenation method for each visible sectors separated by 20° degrees that we appended with all-zero vectors for the non-visible sectors (see, Figure 2 below and Equation 8 in the Appendix).

#### 3.9.3. Normalization and lifetime sparsity

In some experiments we normalized the inputs to unit ℓ_2_ norm for each *d* = 150 dimensional components, provided that at least one of the components differed from zero and dropped the input if all the components were zeroes. This is the “normalized case.” We used spatial sparsification with *k* = 1. We also used lifetime sparsification. The dimension *Q* of the feature map of the autoencoder was set to 30 and we used probabilities of $p=\frac{100}{Q}\%=3.33\%$ and *p* = 6.66%. The *p* = 3.33% means that any component was active once on the average in the sample, but either none of them, one of them, or more than one of them may have assumed non-zero values for an individual input. The all zero case was dropped and thus the average probability was somewhat higher than *p* = 3.33%. The ratio of dropped inputs was smaller for probability *p* = 6.66%.

Concerning the error of the autoencoder we had two options: (a) error of the full output and (b) error only on the visible components that belonged to the viewing angle as in Equation (9). This latter is called masked experiment. We experimented with 3 and 5 layer autoencoders, with the middle layer representing the latent variables. For the 5 layer case, the sizes of the hidden layers were spaced linearly between 2700 and 30 giving rise to layers of dimensions 2, 700, 1, 335, 30, 1, 335, 2, 700 from input to output, respectively.

#### 3.9.4. Magnitude ordering

For each point in the arena we ordered the activity vector's components according to their magnitudes, with the largest being the first. Although the dimension of the representation remains, the individual indices of the place cells disappear: one doesn't know, which place cell has the largest activity, which one is the second largest, and so on. Nonetheless the largest activity will change along straight paths since between two place cell dumps there is always a hump. The oscillation is the basis of learning. Magnitude ordered activities along straight paths may provide information about displacements along the path, since the differences of the magnitudes change. Exceptions correspond to different positions that have the same set of activity magnitudes, which may occur for regular lattices and along lattice translation vectors.

#### 3.9.5. Prediction along straight paths

We performed the prediction experiments on a place cell activity model trained by the autoencoder with a specific set of parameters: we used *p* = 6.66% lifetime sparsity with normalized input and masked loss function with a 220° viewing angle. The model was trained for 100 epochs. We discretized the arena to a 150 × 150 grid and collected place cell activities using the model from each of the 151 × 151 = 22801 grid points for all 18 directions.

We collected data in each direction separately. Distance between the steps equals the grid step distance of the discretized arena. In the learning phase we used *n* = 60, 80, 100 samples of the *m*(= 30) magnitude ordered place cell activities from a *n* step length straight path as inputs. For each step the sample of the closest grid point was taken. The *m* dimensional data sample of the (*n* + 1)^*st*^ step along the same path was used as supervisory predictive information. All sample paths where the necessary *n* + 1 steps doesn't lead out from the arena were used during training.

With this method we can estimate the representation beyond the arena from an initial series of samples by using the predicted estimation for shifting the *n* consecutive samples and dropping the last one. The short distances between the steps aim to imitate gamma-wave sampling.

The software used in these studies can be downloaded from GitHub^1^.

## 4. Results

**Figure 3:**
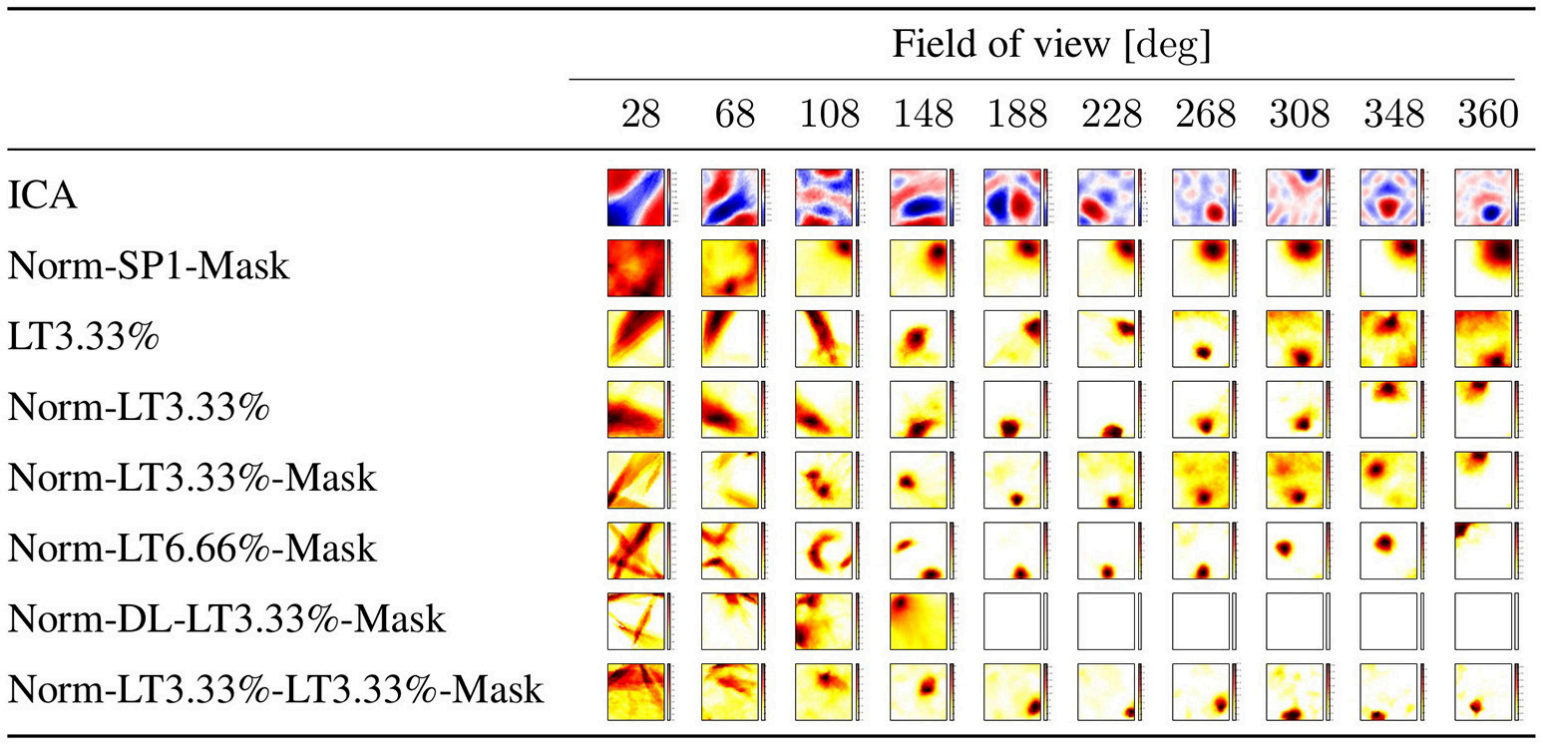
**Linear responses of individual latent units selected randomly: we chose neuron with index 2 from the latent layer**. ICA: values may take positive and negative values. Other experiments: all units are ReLUs, except the output, which is linear. Color coding represents the sum of responses for all directions at a given point. SP1: spatial sparsity with *k* = 1, LT3.3%: lifetime sparsity = 3.3%, Norm: for each 150 components, the ℓ_2_ norm of input is 1 if any of the components is non-zero, Mask: autoencoding error concerns only the visible part of the scene (i.e., the non-masked part of the input) DL: dense layer. “Norm-LT3.3%-LT3.3%-Mask” means normed input, masked error, 5 layers; the input layer, 3 layers with LT sparsity of 3.3% and the output layer. Columns correspond to masks of different angular extents separated by 20° and covering viewing angle of 28°, i.e., they overlap. Left column: a single viewing angle is non-masked. Other columns correspond to 3, 5, …17, 18 non-masked directions in increasing order to the right. (Lőrincz et al., 2016 with permission).

**Figure 4:**
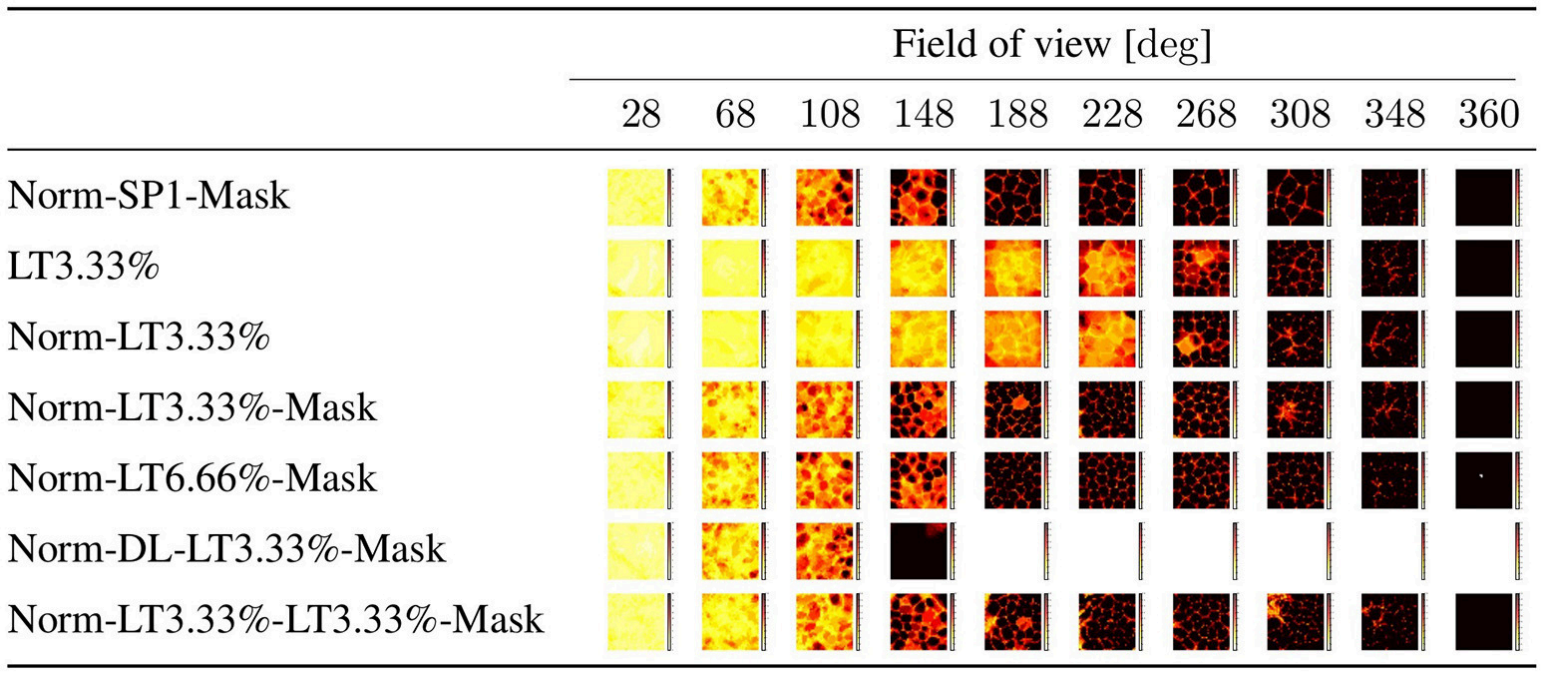
**Angle independence**. Notations are the same as in Figure 3. The highest activity (winning) unit was selected for each input at each position in each direction. We counted the number of wins at each position for each unit and selected the largest number. Results are color coded. Black (18): there is a single winner for all angles at that position. White (0): no response at that point from any neuron in any direction. Values between 1 and 17: the darker the color the larger the direction independence for the best winner at that position. Rows represent different algorithmic components. SP1: spatial sparsity with *k* = 1, LT3.3%: lifetime sparsity = 3.3%, Norm: for each 150 components, the ℓ_2_ norm of input is 1 if any of the components is non-zero, Mask: autoencoding error concerns only the visible part of the scene (i.e., the non-masked part of the input), DL: dense layer. “Norm-LT3.3%-LT3.3%-Mask” means normed input, masked error, 5 layers; the input layer, 3 layers with LT sparsity of 3.3% and the output layer. Columns correspond to masks of different angular extents separated by 20° and covering viewing angle of 28°, i.e., they overlap. Left column: a single viewing angle is non-masked. Other columns correspond to 3, 5, …17, 18 non-masked directions in increasing order to the right. (Lőrincz et al., 2016 with permission).

First, we review our recent results on place cells derived in Lőrincz et al. (2016) and in Lőrincz (2016) for the sake of argumentation and clarity. These results are reproduced in Figures 3, 4, and in Table 1. Then we derive new features related to the place cells. This subsection is followed by the description of our new results on oriented grid cells. They, together, form the Cartesian Factor.

**Table 1:** **Dead neuron count: number of non-responsive computational units**.

	**Field of view [**deg**]**									
	**28**	**68**	**108**	**148**	**188**	**228**	**268**	**308**	**348**	**360**
Norm-SP1-Mask	2	0	5	5	10	12	16	18	15	18
LT3.33%	0	0	0	0	0	2	2	6	8	9
Norm-LT3.33%	0	0	0	1	1	3	2	4	9	11
Norm-LT3.33%-Mask	0	0	0	0	0	0	1	2	7	11
Norm-LT6.66%-Mask	0	0	0	0	0	0	1	4	13	13
Norm-DL-LT3.33%-Mask	0	3	1	29	30	30	30	30	30	30
Norm-LT3.33%-LT3.33%-Mask	0	0	0	0	0	0	0	0	0	0

We note that uniformly distributed inputs and sparsification favors similarly sized sets of the input space, since latent units are competing for responses as we shall discuss it later. Competition gives rise to close packing. In 2D, the locally closest packing is the hexagonal structure and this arrangement is commensurate with the 2D space, so locally close packing can be continued and gives rise to a regular global structure, the triangular lattice. Our arena is, however, a square structure and has 90° symmetry, which is incommensurate with the hexagonal structure. In turn, we expect a close to hexagonal PC structure with reasonable amount of structural errors. Notably, self-supervised predictive compression gives rise to grids and emerging grids show improved hexagonal symmetry and tend to correct the errors of the place cells. Note that the larger the arena, the smaller the effect of the boundary is.

### 4.1. Cartesian abstraction yields place cells

The dependencies of the responses in the hidden representation vs. space and direction are shown in Figures 3, 4, respectively. Linear responses of randomly selected latent units for different algorithms are depicted in Figure 3, illustrating the extent that the responses became localized even in the absence of competition after learning.

Figure 4 shows the direction (in)dependence of the responses. This figure has a special coding method: for each position and for each direction we computed the responses of all 30 neurons of the middle layer of the autoencoder and chose the one with the highest activity. In the ideal case a single neuron wins in all directions at a given position. Therefore, for each position we selected the neuron which won in the most directions (out of the 18) and assigned the number of its winnings to that position. Then we colored each position within the arena with a color between white, when the number is zero, i.e., none of the neurons is responding in any of the directions, and black, when the number is 18, i.e., the winner is the same neuron in all directions. Middle values between 0 and 18 are colored from light yellow to dark red in increasing order. Figure 4 depicts results for different masks. The first column from the left is the case when only a single direction is not masked. Other columns from left to right correspond to cases when 3, 5, …18 directions are not masked.

One should ask (i) if the linear responses are local and activities far from the position of the peak activity are close to zero; (ii) if the number of dead latent units is small, (iii) if responses are direction independent, that is, if we could derive the discretization of space in allothetic coordinates. We found that spatial sparsity with the 3 layer network rendered the output of some or sometimes all hidden units to zero (Table 1). The same happened for the 5 layer network with dense 2*nd* and 4*th* layers and sparse 3*rd* layer. On the other hand, lifetime sparsity *p* = 3.33% with the 5 layer network produced excellent results. Lifetime sparsity *p* = 6.66% also produce high quality PCs. Figure 4 shows that including the mask, direction-invariant activations start to develop at around about 100° (see the second and the third lines), whereas without the mask, similar activations appear at around 230°. For the sake of comparison, we also provide the ICA responses in Figure 3.

### 4.2. Place cells assume close to hexagonal structure

**Figure 5:**
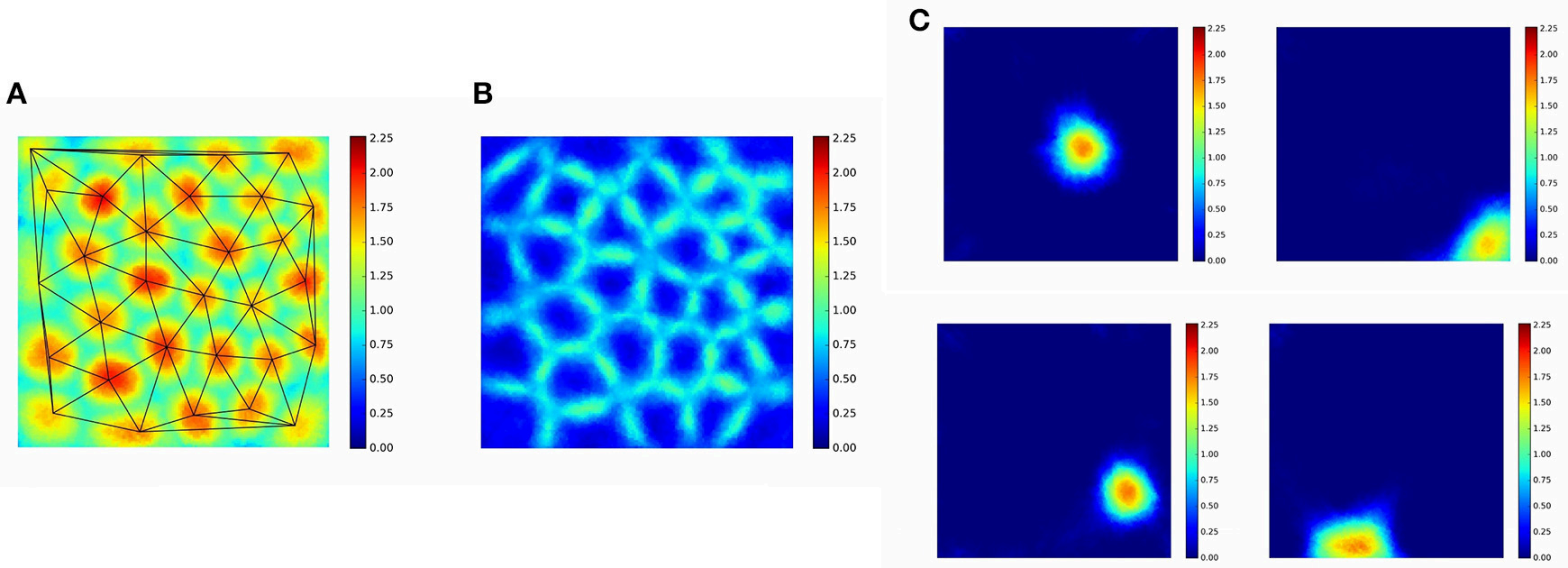
**PC positions make close to hexagonal structure constrained by the non-hexagonal form of the “arena.” (A)** Delaunay triangulation on the linear activities of the first (largest) component of the magnitude ordered representation. **(B)** Linear activities of the second(-largest) component of the magnitude ordered representation. **(C)** Individual PC activities. For more details, see text.

Competition, as it was mentioned above, gives rise to hexagonal close packing in two dimensions, that is in a triangular lattice structure. In our experiments the symmetry is frustrated by the squared boundary of the “arena.” The Delaunay triangulation of Figure 5A shows a number of distorted hexagons, some heptagons, pentagons and—closer to the edges of the “arena”—a few quadrilaterals, too. The more dark red the color, the smaller is the winning domain of the neuron. Sizes are more similar and shapes are more circle-like in the internal part of the “arena,” whereas they are more distorted around the edges and at the corners. The size of the PCs are similar or larger at around the edges and the corners (Figure 5C). The paper written by Muller et al. (2002) reviews the different variables of sensory information that affect the sizes and the densities of PCs. We note that in the experiments, the bags are almost empty at the edges (in 180°) and in the corners (in 270°).

### 4.3. Predictive methods can form grid cells from place cells

**Figure 6:**
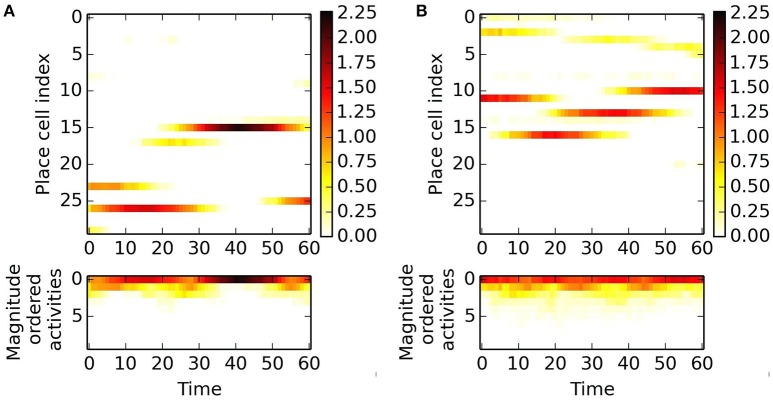
**Magnitude ordered examples at two different positions in two different directions**. Activities are color coded. **(A)** 1st place and 1st direction. **Top**: activities of place cells along a 60 step paths, **bottom**: magnitude ordered activities. **(B)** Alike **(A)**, but for 2nd place and 2nd direction. Different place cells fire. About four place cells produce non-negligible outputs in both cases.

We use pseudoinverse and PLS regression methods to predict the next activity based on a series of previous ones. These methods work on magnitude ordered series and thus they are not associated with individual place cells. Magnitude ordered activities show oscillations along straight paths as shown in Figure 6. Such behavior suits prediction.

We show results for this two linear methods below.

#### 4.3.1. Prediction outside of the “arena”

**Figure 7:**
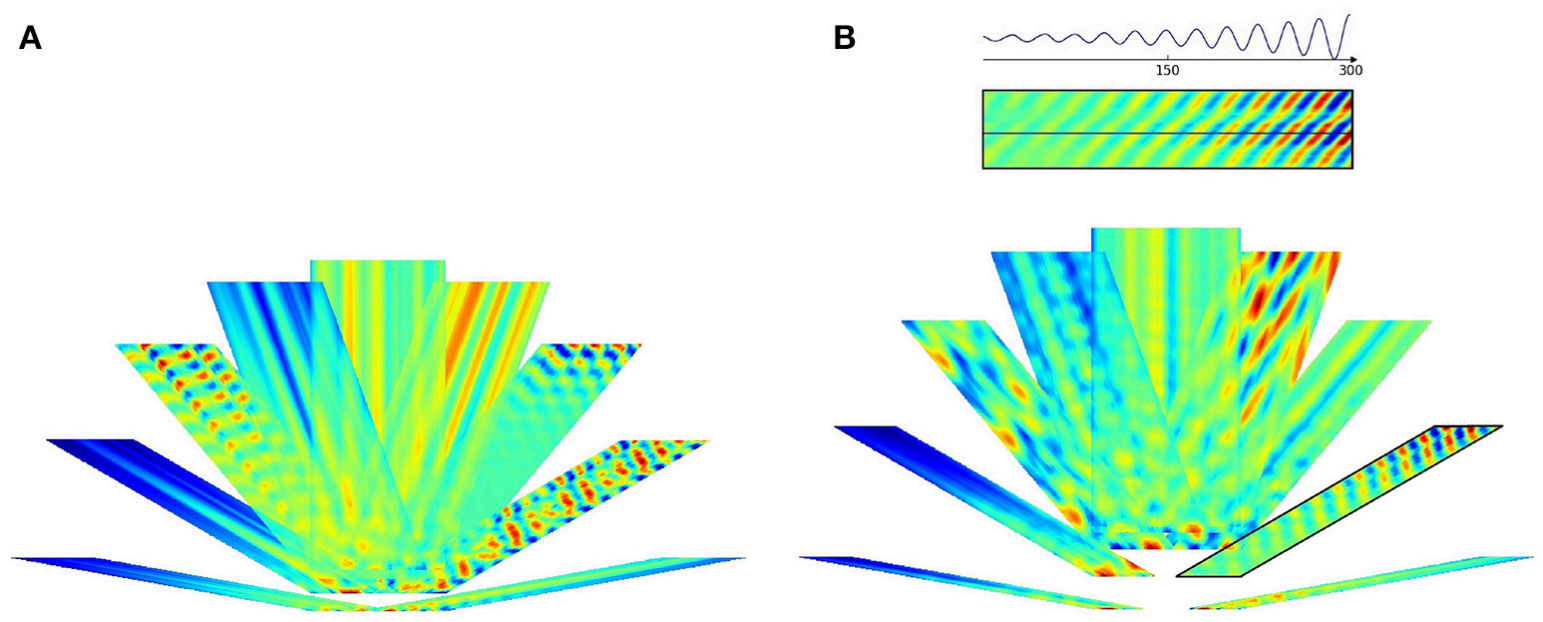
**Pseudoinverse predictions along straight paths beyond the “arena.”** Inset of **(B)** and the parallelogram framed with black line in **(B)** show how the figures were created: oscillating paths along a straight line are color coded and numerous parallel lines were computed. Different directions are cumulated into different parallelograms oriented according to the path traveled. Predictions start from the points of the lower edge of the “arena,” have **(A)** 60 and **(B)** 100 time steps measured in different directions and proceed in that direction. Each direction had its trained predictive matrix that predicted activities from all neighboring oriented lines. Ideal activity pattern forms a triagonal lattice structure. The predictive matrix learned some characteristics of the displacements in the structure, but the prediction is poor.

**Figure 8:**
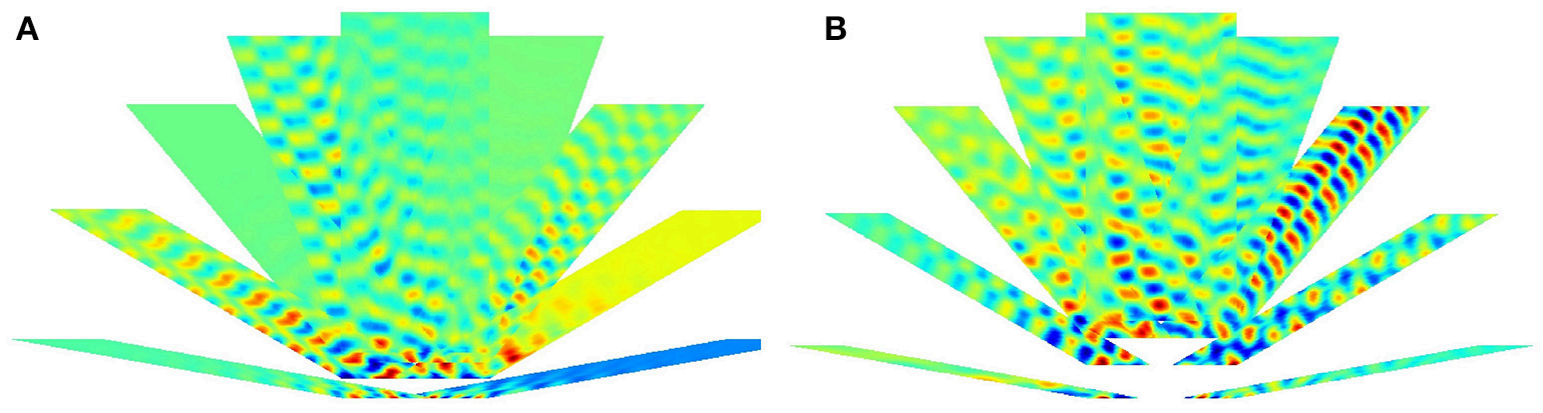
**PLS regression based predictions along straight paths beyond the “arena.”** The subfigures are created alike to those of Figure 7. Predictions start from the points of the lower edge of the “arena,” have **(A)** 60 and **(B)** 100 time steps measured in different directions and proceed in that direction. Each direction had its trained predictive PLS algorithm. The predictions can represent displacement information along the parallel lines and periodic, approximately hexagonal structure appear in a number of directions.

Figures 7, 8 depict the results for the pseudoinverse method and for PLS regression, respectively

**Figure 9:**
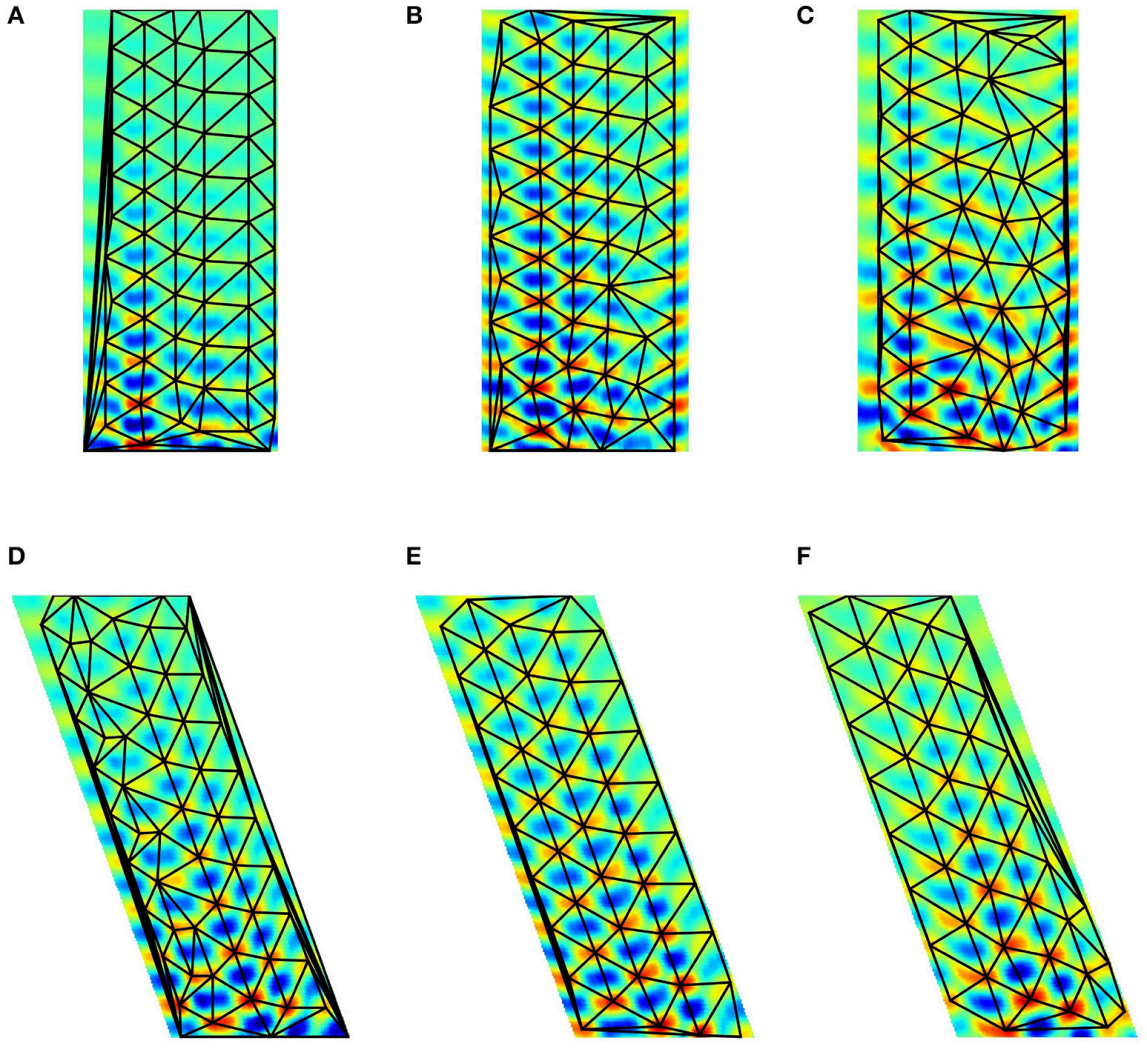
**Delaunay triangulation fitted to the predicted structure from models. (A–C)** Trained on 0° paths **(D–F)** trained on 340° paths. Training paths are 60, 80, and 100 steps long for **(A)**+**(D)**, **(B)**+**(E)**, and **(C)**+**(F)** subfigures, respectively.

PLS regression is a better predictor than the pseudoinverse method. We show predictions starting from a straight line along different directions. Both methods produce results that depend on the position along the starting line. PLS also predicts periodic changes along the paths and this structure is close to hexagonal beyond the “arena”: pentagons and heptagons or other non-hexagonal polygons are rare except around the edges of the predicted region (Figure 9). Predicted signal fades in most cases as prediction proceeds.

Figure 9 show predicted structures at angles 0° (Figures 9A–C) and in 340° (Figures 9D–F), respectively. Prediction takes past values of 60, 80, and 100 steps, respectively (see Figure 9). Outside the arena the number of predicted steps are in the order of 200. Note one step is very small compared to the PCs. If the size of the PCs is about the size of the rat, then the steps are about one twentieth of the rat's size.

For 0°, hexagonal structure is the best for 60 steps, but it fades quickly. Fading decreases for 80 steps, but the structure inherits the PC errors of the arena. This is more so for 100 steps. The case is somewhat different for predictions along 340°. In this case, fadings are similar. Visual inspection says that it is the smallest for the 80 step case. Hexagonal structure is relatively poor for 60 steps and is considerably better for 80 and 100 steps.

The figures demonstrate that close to hexagonal predictions can arise. The following notes are due here. The more the information from the past, the more the squared arena frustrates the hexagonal structure. Different directions approximate hexagonal structure differently, depending on the error structure within the squared arena. We also note that the ratio between length of the boundary and the size of the arena decreases the frustrating effect of boundary as the size of the arena increases.

From the point of view of model categories, the predictive network that uses its own output to complement (increment) its own input is an *attractor network*.

## 5. Discussion

First, we review and discuss the general and specific features of our results. We also link them to the neural substrate and consider the computational potentials from the point of view of semantic memory, episodic memory, and reinforcement learning.

### 5.1. General considerations

Our goal was to find hidden and abstract Cartesian Factor, that is, the discretization of the factor and the related attractor network that serves as an implicit representation of the related metric, provided that we have the complementing one. The method is general. We applied the approach as a model for the EHC. We assumed that we are having the head direction cells. From the point of view of the neuronal computations, attractor models working on set of cells are the most promising (see e.g., Skaggs et al., 1995; Redish et al., 1996 reviewed by Clark and Taube, 2012).

From the theoretical point of view, the abstraction that we want to develop is similar to geometrical abstractions or algebraic abstractions: they cannot be sensed directly, so they are latent. They are also Cartesian in the sense that they are like coordinates in an abstract space. In turn, they enable highly compressed descriptions. According to our assumptions, Cartesian Factors are low dimensional and only a few of them are needed for the mental solving of certain tasks and for the execution of decisions. Such elimination of variables is critical for reinforcement learning (Kearns and Koller, 1999; Boutilier et al., 2000; Szita and Lőrincz, 2009). The example in the context of navigation is path planning. Path planning can be accomplished in a discretized allothetic abstraction independently from idiothetic visual observations. This property lowers computational needs considerably. In turn, optimization of problem solving depends on the capability of forming low dimensional Cartesian Factors that are relevant for planning.

The concept of Cartesian Factors is closely related to Gestalt principles. Gestalt psychologists considered objects as perceived and as global constructs made of the constituting elements *within an environment*. Gestalt psychology has a number of concepts or laws on how to group things or events. Among these are the *Law of Proximity* and the *Law of Continuity*: according to Köhler (1929), “what moves together, belongs together” (see e.g., Paglieri, 2012 and the references therein). Self-motion, for example, allows the separation of the self from the rest of the environment and can be uncovered by temporal information. Such information drives the SFA procedure explored by Wiskott's group (Franzius et al., 2007; Schönfeld and Wiskott, 2015). They found that in realistic conditions and for large viewing angles, direction independent place cells can be formed by means of the temporal information. However, temporal information may be limited due to sudden environmental changes or occlusions. Furthermore, limiting the algorithm to temporal information limits the Gestalt principles to a few of them.

Another Gestalt principle is the *Law of Similarity*. This principle does not rely on temporal information and could be more adequate for general databases. Our algorithms implicitly exploit this principle through the concatenated input pieces that correspond to different viewing directions and may have identical, similar or very different information contents, subject to the position and the orientation. In our work, we used head direction and idiothetic information. The idiothetic observation was in the form of a *bag model*. Bag models are widely used in natural language processing, called the *bag of words* (BoW) representation, and in image processing, called the *bag of keypoints* (BoK) representation in this case. It means that we have access to the components being present at a time, but not about their order in time or space. In other words, the bag model is similar to the *what system* of visual information processing, described first by Mishkin and Ungerleider (1982).

Considering the bag model from another point of view, any component in the bag requires an invariant representation. For BoW, stemming is the tool. BoK can be based, for example, on local scale invariant features introduced by Lowe (1999). Whereas stemming eliminates the details and becomes invariant of the syntax, scale invariant features incorporate scale and rotation variations in order to become invariant to transformations. The case of PCs is similar, their outputs are invariant to directional changes. In turn, our concept can be formulated as follows: we assume that beyond having a Cartesian Factor, (a) some “details,” such as suffixes or scaling and rotations or orientation, can be measured, (b) the bag model has been built and the “suffixes” are either explicitly embedded into the complementing observations (i.e., into BoK) or neglected (i.e., from BoW), (c) the complementing observations hide a low dimensional space and thus it can be discretized with limited resources, and (d) this low dimensional space may have a related metric. In the case of documents, discretization may correspond to topics and the underlying structure is similar to a tree, since each topic may have subtopics. In the case of scale invariant features, the complementing space is the space of shapes and textures and it is very large. However, if the bag of environmental visual cues can be formed as we did here, then it can support the discretization of the environment as we showed in our computer studies.

We should note that similarity based grouping is an alternative to temporal grouping and can be used if the latter is not available. For example, temporal grouping is impaired in akinetopsia, but the representation of the 3D world is not impaired. It seems reasonable to expect that temporal and similarity based algorithms *together* learn faster, perform more robustly and more precisely, e.g., if the task is forecasting.

The novelty of our contribution is the concept of Cartesian Factor. Such factors can be developed in many ways. Here, we put forth a similarity based algorithm, studied it, and suggest to unify it with other Gestalt principles. From the point of view of Gestalt theory, the novelty in this work is that we are looking for descriptors of the global context, that is, the environment itself. Compression takes place via sparse autoencoding, when encoding is based on the information that we apply via *masking* part of the input representation. Note that the input is in the form of a *bag representation*, which is a sufficient condition here.

We added temporal clues and developed predictive systems using pseudoinverse computations and PLS regression. Pseudoinverse computation seem to fit the structure of the superficial layers of the entorhinal cortex (Lőrincz and Szirtes, 2009) and the non-linear extensions are feasible. For pseudoinverse computation and for PLS, we found that PLS regression can provide more regular predictions. Furthermore, we found that the oriented hexagonal-like structures continued beyond the observed “arena” can keep the hexagonal regularity, sometimes to a better extent than the original set of PCs learned in a non-hexagonal environment. We suspect that the highly precise hexagonal grids (see e.g., the review written by Buzsáki and Moser, 2013 and the cited references therein) may emerge by including an interplay between the PCs and the oriented grids when orientation free grids are developed, since the trigonal grid is the common structure in the different directions.

### 5.2. Cell types developed

Using the bag model, we could develop place cells by covering viewing angles of about 100°. Further improvement can be expected if (i) deeper networks are applied and (ii) if temporal changes are included. We found in our simulations that sparsity should be kept for deeper networks at least for some of the layers. No experiments were conducted on pixel based visual information, a much higher dimensional representation that has pixel-wise nonlinearities. Such nonlinearities can be overcome in many ways, including temporal methods as demonstrated by Franzius et al. (2007) and Schönfeld and Wiskott (2015). An extension of our architecture to a hierarchy may also suffice.

While the first largest amplitude PC signal must belong to the closest cell, the second largest must belong to its nearest neighbor along the path. In turn, second largest amplitudes should uncover the Voronoi tessellation of the PCs as demonstrated in our computer experiments (Figure 5B).

From the algorithmic point of view, when a path proceeds toward the border of the “arena” and gets close to it, the second largest component becomes very small, since there is no cell beyond the border and the second nearest neighbor can be far at the sides. Assume that a cell responds to the ratio between the largest activity and the second largest one. This cell will show high activity when the path is directed toward the border and the position is close to the border, since the second largest activity belongs to a remote PC and is small. This cell would behave alike to *border cells* even in dark. We should note that according to the long held view, interneurons approximate arithmetic operations, such as *subtraction, division* or *shunting* of the excitation.

By means of PCs, we could develop oriented grid cells and could derive some precursors for border cells. Three simple and justifiable algorithmic operations were exploited, (i) the integrate-and-fire mechanism, (ii) features of the theta waves, and (iii) a self-supervisory compression in the form of pseudoinverse computation and PLS regression. Self-supervision means that actual signals supervise delayed signals during learning. Magnitude based ordering may occur in the neural substrate, e.g., if magnitudes are converted to time giving rise to time ordering. However, some kind of clock is needed for telling the zero instant of the ordering. Intriguingly, the phase of theta wave can play the role of such a clock. Indeed, during the first half of the theta cycle, cells that fire represent current position, whereas during the second half of the theta cycle temporally ordered (future) place cells fire (Sanders et al., 2015). These findings point to a more complex mechanism: cells that represent the past cant fire in the second half of the theta wave. We used a concatenation mechanism for prediction and, in turn, our model suggests a predictive learning mechanism that overbridges theta cycles and exploits the activities of the second halves of the theta cycles.

Recent results from Ferrante et al. (2016b) show that different functional groups of pyramidal and inhibitory neurons are present in the entorhinal cortex. Such groups may satisfy our constraints that magnitude based ordering can support oriented grid cell formation via self-supervised prediction as well as border cell formation via shunting inhibition. Here is putative model for the latter. Consider the integrate-and-fire model. Spikes that come first excite the neuron and if delayed spikes that respond to the second largest activities are not capable for the ignition of shunting inhibition—e.g., if the animal is close to the border and no PC is in that direction—then the cell will fire and the cell will behave like a border cell. The head direction dependence is, however, more complex as reported by the original work of Solstad et al. (2008) calling for more detailed models based on sophisticated features, see e.g., the review of Kepecs and Fishell (2014) and the papers of Ferrante et al. (2016a), being outside of the scope of this paper.

### 5.3. Order of learning in the model

We used HCs for learning PCs without temporal information. We developed oriented grid cells from the PCs by means of temporal information and self-supervised compression. We showed that prediction becomes more regular (more hexagonal-like) if it is continued beyond the area represented by PCs. Temporal information on the second largest amplitudes gives rise to the Voronoi polygons on the set of PCs and may uncover border responses, e.g., by insufficient shunting inhibition. This algorithmic feature remains valid in dark, since it relies on the available set of PCs.

Other entorhinal cell types, such as speed cells and direction independent grid cells pose further challenges for our model. Speed cells described by Kropff et al. (2015), can be easily formed, since the firing rate of oriented grids is a monotone function of speed as found by Sargolini et al. (2006). For example, the max pooling operation, being well documented for the primary visual cortex (Movshon et al., 1978; Mechler and Ringach, 2002; Touryan et al., 2005), suits the needs. The idea can be traced back to the work of Fukushima (1980) and has gained attention from the point of view of (i) invariant representations (Serre et al., 2002), (ii) as a tool for efficient feature extraction, and (iii) reduction of the dimension of the representation (Huang et al., 2007). From the point of view of grid cells, a max pooling neuron outputs the largest activity and thus it loses orientation and displacement dependencies making the activity a monotone function of the speed.

The model of direction independent grid cells is more challenging, since there are additional constraints: firing should be continued (a) at any point, (b) including the absence of learned PCs, and (c) according to the displacement of the grid in any changes of the direction. A number of neurally plausible models based on different assumptions have been built see, e.g., the works of Burgess and O'Keefe (2011), Giocomo et al. (2011), and Kesner and Rolls (2015) and the cited references. The capability for planning, however, seems crucial as emphasized by Buzsáki and Moser (2013) and Sanders et al. (2015). It has been included into a detailed model by Sanders et al. (2015). Compared to these model, the Cartesian Factor principle is a high level description that aims to shed light onto the origin of the key algorithmic building blocks of the development of neural representations.

The Cartesian Factor principle suggests the following order of learning: (i) head direction cells, (ii) place cells, (iii) oriented grid cells, (iv) direction free grid cell representation by means of an interplay between place cells and grid cells. According to the recent paper from Rowland et al. (2016), there are two possible routes for grid cell formation: it is either species specific or spatial experience shapes the grid system. Our model proposes the latter option and fits the experimentally found order of learning reviewed in the cited paper.

We illustrated that the hexagonal like symmetry of the grid cells can be maintained in the absence of information form PCs. Planning and then traveling along loops, e.g., exploring and then homing, can serve the tuning of the grid cells. It may be worth noting that both grids and PCs change under slight distortion of the “arena” showing the coupling between these representations.

Along the same line of thoughts, our model is based on an autoencoder, which—by construction—is also a comparator (Lőrincz and Buzsáki, 2000) as suggested for the hippocampal function by Vinogradova (2001) and others, see the cited references. In the autoencoder, the input received is compared with the representation generated output. In case of mismatch, the adjustment of the representation may take place and the same error may drive Hebbian learning. Such error based optimization of the representation and learning were suggested by Lőrincz and Buzsáki (2000) and Chrobak et al. (2000) and elaborated by Lőrincz and Szirtes (2009).

Our sparse autoencoder hypothesis is supported by the fact that activity patterns are very sparse in the CA1 subfield of the hippocampus. We found in our numerical experiments that two stages are needed for the development of sparse representations, one for real time processing that uses spatial sparsity, and another one for off-line processing, when replayed inputs satisfy lifetime sparsity constraints. Such differences may show up in statistical evaluations of theta phase patterns and SPW-R patterns, with the former representing the actual path, whereas the latter may perform lifetime sparsification. However, behavioral relevance may modulate this simple picture.

### 5.4. Special features of the algorithms

The particular features of our algorithmic approach are as follows:

1. Sparse autoencoding requires two stage operation, one for real time and another one for learning. The latter should implement or approximate lifetime sparsity. Imperfect lifetime sparsity may give rise to silent neurons not responding to inputs. Homeostasis can counteract this process, enabling an adjustable reservoir of PCs for learning new information. Homeostatic maintenance of the activity may manifest itself through low spatial specificity. Such neurons have been found by Grosmark and Buzsáki (2016), but the picture seems more sophisticated.
2. Temporal ordering is necessary for the predictive compression in our model. This is the core step that sets the high-level grid representation free from external observations. Theta-waves or integrate-and-fire behavior, possibly both, are candidates for temporal ordering.
3. The bag model simplifies both the algorithm and representation; it decreases the dimensionality of the input and neglects many of the details. It keeps track of the components, but not their actual manifestations. The bag representation is analogous of the “what system” that has information about the objects present, but not about their positions, for example. From the point of view of component based representation, the bag model resembles to the “recognition by components” principle put forth by Biederman (1987) for visual inputs.
4. The model of Cartesian Factor formation needs neurons that can multiply and can produce conjunctive representations, e.g., between the visual cues and the head direction cells. Candidates for such computations include (i) the logical operations, such as the AND operation made possible by coincidence detection (for a recent review, see the work of Stuart and Spruston, 2015), (ii) the interplay between distal and proximal dendritic regions—when the proximal input enhances the propagation of the distal dendritic spikes—can also support a multiplicative function (Larkum et al., 2001; Jarsky et al., 2005). We note that the EHC has sophisticated interconnections between distant and proximal regions (Gigg, 2006). We exploited the multiplicative feature in our representation by using the product space and zero some of the inputs by (multiplicative) masking.

### 5.5. Relation to meta-level cognition

Cartesian Factors select features of the world and a limited set of features may be sufficient for solving distinct problems. Path planning is an example. The grid like structure, its potentials for path planning and distance estimation as described in Huhn et al. (2009), for example, are high level descriptors of the world. They tell very little about the actual sensory information. The autoencoding principle can serve both functions that is (i) the manipulation at the meta-, or symbolic level, such as the computation of distances on the grid structure and (ii) the low level input-like representation via the estimations of the inputs or the inputs that follow. The autoencoding principle resolves the homunculus fallacy by saying that “making sense of the input” is the function of the representation that approximates the input (Lőrincz et al., 2002). We undersign the view that the estimation of the input occurs via hierarchical bag representations that neglect more and more details bottom-up and combine more and more (Cartesian) factors top-down. One may say that in the top-down generation of the estimated input, meta level description becomes semantically embedded by means of the contributing Cartesian Factors.

One can also treat episodic memory in the context of the autoencoding principle. The appearances or the disappearances of sparse codes by time can be seen as starting and ending points of events. Such description fits factored reinforcement learning (Szita et al., 2003). Taken together, our algorithms and the concept of Cartesian Factors can provide simple clues about the working mechanisms of the “cognitive map” in such a way that the computations avoid combinatorial explosions (Szita and Lőrincz, 2009) and thus escape the curse of dimensionality, explicated by Bellman (1958).

## 6. Conclusions

We put forth the novel concept of Cartesian Factors. The working was demonstrated by forming of place cells and grid cells, where we exploited the complementary information, the head direction cells. Our proposed cognitive mechanism does not work in the absence of such information. We note that upon destroying the vestibular system, which is critical for having head direction cells, no place cell is formed (Taube, 2007; Winter and Taube, 2014).

Our algorithm is a sparse autoencoding mechanism that can be deep, but should be sparse in the hidden layers according to the numerical studies. Our algorithm relies on the *bag model* that we related to the *what system*. The bag model works with a collection of input portions that represent the same quantity type, or object types, or episode types, such as idiothetic inputs collected at the same position but in different directions, or the different views of an object, or the different temporal variations starting from a given state and ending in an other one, respectively. The different mechanisms should support each other.

The particular feature of the Cartesian Factors is that a few of them may be sufficient for solving cognitive problems. An example is path planning on the “cognitive map” if neighbor relations are available. Elimination of directions from the path planning problem reduces the state space in the exponent. This is a very important advantage in decision making.

We used the discretized form of the Cartesian Factors to develop the (implicit) metric-like representation that can be continued beyond the experienced portion of the factor. The self-supervised predictive compression method was illustrated in oriented grid formation. We found that the predicted grids can be very regular and may compensate for the errors of the underling discretization of the factor. We used magnitude based ordering and suggested integrate-and-fire mechanism and theta wave based firing as candidate mechanisms for this learning stage. The attractive feature of magnitude ordering is that it detaches sensory information from the underlying (metrical) structure and enables extrapolation beyond the already observed part of the world.

The interplay between (a) the detachment of the direct sensory information, (b) the manipulation in the underlying space, and (c) the association of new sensory information to the extrapolated structure, in other words, the separation of grids from visual sensory information, the prediction on the grids can be seen as symbol learning, symbol manipulation, respectively. The association of grid cell activities to visual information, on the other hand, corresponds to symbol grounding in our framework and offers a solution to the grounding problem targeted first by Harnad (1990).

We found that the concept of Cartesian Factors approximates well the learning order and impairment related features of head direction cells, place cells, and oriented grid cells. The concept also provides hints about border cells that can fire in the absence of visual information. We argued that border cells, direction free grid cells, and speed cells can emerge in the model via neurally plausible mechanisms, but they require further studies.

In sum, the concept of Cartesian Factors offers (a) a solution for the curse of dimensionality problem of reinforcement learning, (b) an explanation for a number of features of the EHC, such as sparse representation, distinct cell types, and the order of learning, (c) a framework for symbol formation, symbol manipulation, and symbol grounding processes, and (d) a mechanism for the learning of attractor models by means of magnitude ordering.

## Author contributions

The main contributions of AL cover the basic concepts, including the idea of Cartesian Factors, the relations to the cognitive map, the grid structure, and other cell types, the connections to cognitive science, and factored reinforcement learning. Computer studies, including some discoveries from computation based modeling are the key contributions of AS. They contributed equally to the design of the work, the analysis, and the interpretation of data. The paper was written jointly with figures being produced mostly by AS, whereas writing is mostly due to AL.

## Funding

This research was supported by the EIT Digital grant (Grant No. 16257).

## Acknowledgments

We are grateful to the reviewers for their helpful comments and suggestions.

### Conflict of interest statement

The authors declare that the research was conducted in the absence of any commercial or financial relationships that could be construed as a potential conflict of interest.

## Footnotes

^1^ https://github.com/asarkany/ehcmodel

## Appendix: details of the algorithmic formulation of Cartesian Factor learning

Assume that a latent random variable *Z* and an observed random variable *Y* are continuous and together they fully explain away another observed binary random variable *X*. The ranges of *Z* and *Y* are supposed to be grid discretized finite *r*- and one-dimensional intervals, respectively. We denote the resulting grid points by (*z*^(*m*)^, *y*^(*l*)^) ∈ ℝ^*r*^ × ℝ; *l* = 0, …, *L*, *m* = 1, …, (*M* + 1)^*r*^, *L, M, r* ∈ ℕ. The indices *m* = 1, …, (*M* + 1)^*r*^ are supposed to be scrambled throughout training (i.e., we assume no topology between *z*^(*m*)^). Then observation ***x***^(*m, l*)^ ∈ {0, 1}^*d*^ is generated by a highly non-linear function *g*:ℝ^*r*^ × {1, …, *L*} → {0, 1}^*d*^ from grid point *z*^(*m*)^ and grid interval [*y*^(*l*−1)^, *y*^(*l*)^) as

(7)
$$\begin{array}{l} x^{\left(m,l\right)}=g\left(z^{\left(m\right)},l\right) \end{array}$$

for *m* = 1, …, (*M* + 1)^*r*^; *l* = 1, …, *L*. For each fixed *m*, one is given masks $V_{i,\cdot }\in {\left\{0,1\right\}}^{L}$; $\overset{L}{\underset{l=1}{\sum }}V_{i,l}=v\in \mathbb{N}$ indexing pairs of the form (*l, **x***^(*m, l*)^), where *i* = 1, …, *I* is a global index. Provided such a sample from *Y* and *X*, we aim to approximate the discretized version of *Z*.

We formulated the above problem as a multilayer feedforward *lifetime sparse autoencoding* (Makhzani and Frey, 2015) procedure with input matrix ***X*** ∈ {0, 1}^*I* × *J*^ utilizing two novelties: concatenated input vectors and a masked loss function are motivated by the input structure. In order to construct the inputs ***X***_*i*, ·_; *i* = 1, …, *I* of size *J* = *L*·*d*, we coupled each *v*-tuple of ***x***^(*m, l*)^ vectors for fixed *m* into a single block-vector using the ***V***_*i*, ·_ values as follows:

(8)
$$\begin{array}{l} X_{i,\cdot }=[\begin{array}{ccc} V_{i,1}\cdot x^{\left(m,1\right)}, & \ldots ,V_{i,l}\cdot x^{\left(m,l\right)}, & \ldots ,V_{i,L}\cdot x^{\left(m,L\right)}\\ ] \end{array}. \end{array}$$

Then, we used the ℓ_2_ reconstruction error as the loss, but on a restricted set of elements, namely, on the *v* non-zero blocks for each input:

(9)
$$\begin{array}{l} l(X,\hat{X},V):=\frac{1}{I}\sum_{\begin{array}{c} i=1,\ldots ,I\\ j=1,\ldots ,J \end{array}}\;V_{i,\lfloor \frac{j-1}{d}+1\rfloor }\cdot {\left(X_{i,j}-{\hat{X}}_{i,j}\right)}^{2} \end{array}$$

where $\hat{X}$ denotes the output of the decoder network. Finally, a sparse non-linearity was imposed on top of each encoder layer, which selected the *k* percent topmost activations across one component. We applied both lifetime (Makhzani and Frey, 2015) and spatial sparsification (Makhzani and Frey, 2013). Multilayer autoencoders with rectified linear units, *k* = 1 spatial sparsity, *p%*-sparse lifetime sparsity, and linear decoder output layer make the non-linear units of the network.

We implemented our method in the Python library Theano (Bergstra et al., 2010) based upon the SciPy2015 GitHub repository^2^.
